# Visceral obesity and the risk of Barrett's esophagus in Japanese patients with non-alcoholic fatty liver disease

**DOI:** 10.1186/1471-230X-9-56

**Published:** 2009-07-21

**Authors:** Tomoyuki Akiyama, Masato Yoneda, Masahiko Inamori, Hiroshi Iida, Hiroki Endo, Kunihiro Hosono, Kyoko Yoneda, Koji Fujita, Tomoko Koide, Chikako Tokoro, Hirokazu Takahashi, Ayumu Goto, Yasunobu Abe, Hiroyuki Kirikoshi, Noritoshi Kobayashi, Kensuke Kubota, Satoru Saito, Atsushi Nakajima

**Affiliations:** 1 Gastroenterology Division, Yokohama City University School of Medicine, 3-9 Fukuura, Kanazawa-ku, Yokohama, Japan

## Abstract

**Background:**

The association between obesity and the risk of Barrett's esophagus (BE) is unclear. Furthermore, the association between visceral obesity and the risk of BE is entirely unknown.

**Methods:**

We conducted a retrospective study in 163 patients with non-alcoholic fatty liver disease (NAFLD) who underwent both endoscopy and abdominal CT at an interval of less than a year at our institution. BE was endoscopically diagnosed based on the Prague C & M Criteria. The surface areas of visceral adipose tissue (VAT) and subcutaneous adipose tissue (SAT) were calculated from CT images at the level of the umbilicus. The correlations between the BMI, VAT, and SAT and the risk of BE were examined by univariate and multivariate analyses.

**Results:**

Sixty-nine of the 163 study participants (42.3%) were diagnosed to have endoscopic BE, which was classified as short-segment BE (SSBE) in almost all of the cases. There were no significant differences in the age or gender distribution between the groups with and without BE. According to the results of the univariate analysis, VAT was significantly associated with the risk of BE; the BMI tended to be higher in the group with BE than in the group without BE, but this relation did not reach statistical significance. VAT was independently associated with the risk of BE even after adjustment for the BMI.

**Conclusion:**

In Japanese patients with NAFLD, obesity tended to be associated with the risk of BE, and this risk appeared to be mediated for the most part by abdominal visceral adiposity.

## Background

Metabolic syndrome is a cluster of metabolic abnormalities consisting essentially of abdominal obesity, especially visceral obesity, and has been highlighted as a risk factor for the insulin resistance syndrome, cardiovascular disease, and other chronic diseases [1,2]. Non-alcoholic fatty liver disease (NAFLD), a spectrum of liver disease ranging from simple steatosis to non-alcoholic steatohepatitis, is increasingly recognized as the hepatic manifestation of metabolic syndrome, and liver inflammation and fibrosis are directly associated with visceral obesity, independent of insulin resistance and hepatic steatosis [3,4].

There has been a marked increase recently in the incidence of gastroesophageal reflux disease (GERD)-related disorders (symptoms, erosive esophagitis, Barrett's esophagus (BE), and esophageal adenocarcinoma) in the United States and western countries [5-7], and this has been paralleled by an increased prevalence of obesity [8,9]. Obesity, as measured by the body mass index (BMI), is one of the strongest risk factors for GERD symptoms, erosive esophagitis, and esophageal adenocarcinoma [10-14], while it remains controversial whether obesity is an independent risk factor for BE. Several studies have examined the association between obesity and BE and inconsistent results have been reported, ranging from a significantly increased risk associated with a high BMI [15,16], significantly increased risk associated with a high BMI only in the presence of GERD symptoms [17], or no association at all [18]. There has been recent interest in the possible role of abdominal obesity in the development of GERD-related disorders. Recent studies have shown abdominal obesity, as defined by the waist circumference (WC), waist-to-hip ratio (WHR) or the surface area of the visceral adipose tissue (VAT) as measured on abdominal computed tomographic (CT) images, as a risk factor for BE independent of the BMI, with the association between BMI and BE being no longer observed after adjustment for the WC, WHR, or VAT [15,19,20]. These studies suggest that abdominal fat might mediate the association of obesity with the risk of BE. In addition to the mechanical effects of abdominal obesity, that is, increase of the intra-abdominal pressure by the presence of a large amount of adipose tissue, circulating factors secreted from the visceral adipose tissue, such as tumor necrosis factor-α(TNF-α), interleukin-6 (IL-6), leptin, and adiponectin, have also been proposed to be pathogenically linked to BE and esophageal adenocarcinoma [21-26]. Although it is possible that visceral obesity, as the core of metabolic syndrome, may predict the risk of BE or esophageal adenocarcinoma better than simple obesity, little evidence exists confirming such associations. To the best of our knowledge, no studies have been conducted until date to examine the effect of visceral obesity, as measured on abdominal CT images, on the risk of development of BE in the Asian population.

In this retrospective study, we examined the effect of simple obesity, as measured by the BMI, and visceral obesity, as measured on abdominal CT images, on the risk of BE in Japanese patients with NAFLD, as the hepatic manifestation of metabolic syndrome.

## Methods

### Patients

One hundred sixty-three consecutive NAFLD patients (83 men and 80 women; median age, 60 years; age range, 23–80 years) who underwent both endoscopy and abdominal CT at an interval of less than a year at the Gastroenterology Division of Yokohama City University Hospital between December 2003 and January 2009 were enrolled in the present retrospective study. NAFLD was diagnosed according to the following criteria: i) slight diffuse increase in bright homogeneous echoes in the liver parenchyma with normal visualization of the diaphragm and portal and hepatic vein borders and normal hepatorenal echogenicity contrast; ii) diffuse increase in bright echoes in the liver parenchyma with slightly impaired visualization of the peripheral portal and hepatic vein borders; iii) marked increase in bright echoes at a shallow depth with deep attenuation, impaired visualization of the diaphragm and marked vascular blurring. The patients were included if they fulfilled the following criteria: i) absence of serological markers of hepatitis B virus infection (HBV surface antigen and anti-HBc antibody) and hepatitis C virus infection (anti-HCV antibody): ii) absence of evidence of autoimmune liver disease or alcoholic liver disease (>20 g of alcohol per day).

The exclusion criteria were: difficulty in obtaining the complete patient profiles from the medical records, refusal of the patient to participate in the study, or a previous history of upper gastrointestinal tract surgery.

### Endoscopic findings

Our hospital operates a digital filing system for endoscopic images. All the digital endoscopic images of the enrolled subjects in this study were independently and retrospectively reviewed by two trained endoscopists to investigate the endoscopic findings, including hiatal hernia, erosive esophagitis, and BE. If there was any inconsistency in the assessment of the digital endoscopic images between the two investigators, the final diagnosis was arrived at a joint review of the digital endoscopic images.

### Hiatal hernia

Hiatal hernia was diagnosed when the distance between the gastroesophageal junction and the diaphragmatic hiatus was 2 cm or more.

### Erosive esophagitis

Erosive esophagitis was diagnosed based on the Los Angeles Classification [27] and was divided into three groups: absent, mild (grades A and B), and severe (grades C and D).

### Barrett's esophagus

The presence of BE was diagnosed based on the Prague C & M Criteria [28]. According to these criteria, BE is defined as the macroscopic identification, using a standard endoscopy exam, of abnormal columnar esophageal epithelium suggestive of a columnar epithelium-lined distal esophagus. The length of BE is measured (in centimeters) using the circumferential extent (the C extent) and maximum extent (the M extent) above the gastroesophageal junction, identified as the proximal margin of the gastric mucosal folds [28].

### Visceral adipose tissue (VAT) and Subcutaneous adipose tissue (SAT)

To quantify the visceral adipose tissue (VAT) and subcutaneous adipose tissue (SAT) area in cm^2 ^using the Fat Scan software (N2 System Corporation, Kobe, Japan), a simple CT scan was obtained at the level of the umbilicus, with an attenuation range of -50 to -250 Hounsfield units. VAT was defined as intra-abdominal fat bound by parietal peritoneum or the fascia transversalis, excluding the vertebral column and the paraspinal muscles, and SAT was defined as fat superficial to the abdominal and back muscles. Using a cursor, the VAT area was then measured around the inner boundary of the abdominal wall muscles. A region of interest (ROI) drawn around the external margin of the dermis was used to calculate the total adipose tissue (TAT) area. The SAT area was obtained by subtracting the VAT area from the TAT area.

### Patient profiles and Laboratory values

We collected information from the medical records on demographic variables, BMI, serum leptin and adiponectin levels, and other laboratory parameters, including serum aspartate aminotransferase (AST), alanine aminotransferase (ALT), cholinesterase (CHE), high-density lipoprotein (HDL), low-density lipoprotein (LDL), triglycerides (TG), and the HOMA-IR levels.

### Ethics

The study was conducted in accordance with the Declaration of Helsinki. The study protocol was approved by the Ethics Committee of Yokohama City University Hospital. All of the enrolled patients provided consent for participation in the study.

### Statistical analysis

In the present study, the risk factors were compared between patients with and without BE. The statistical analysis included a Chi-square test or Fisher's exact test to compare percentages and a Mann-Whitney *U *test to compare continuous data. Various risk factors were also evaluated simultaneously using a logistic regression model. The statistical significance level was set at p < 0.05. All the statistical analyses were performed using the Stat View software (SAS Institute, Cary, N.C.).

## Results

The baseline characteristics of the study population are summarized in Table 1. A total of 34 cases (20.9%) had erosive esophagitis, which was mild (LA Classification grades A and B) in most of the cases (17.8%), and severe (LA Classification grades C and D) in only a few (3.1%) (Table 1). The present study demonstrated that 42.3% of the total study population was diagnosed as having BE based on the Prague C & M Criteria [28]. These cases consisted of 41.1% with short-segment BE (SSBE), whose circumferential (C) extent was less than 3 cm, and 1.2% with long-segment BE (LSBE), whose C extent was 3 cm or more (Table 1). Table 2 shows the results of a univariate analysis conducted to compare the clinical characteristics of the groups with and without BE. There were no significant differences in the age (p = 0.8825) or gender distribution (p = 0.5542) between the two groups. As compared to the group without BE, the group with BE showed higher values of BMI, VAT, and SAT, although only the difference in VAT reached statistical significance (p = 0.0089). The prevalences of hiatal hernia (p < 0.0001) and erosive esophagitis (p = 0.0099) were significantly higher in the group with BE than in the group without BE (Table 2). Multiple logistic regression analysis using clinical factors, including the age, gender, BMI, hiatal hernia, VAT and SAT was conducted to determine the clinical parameters associated with BE (Table 3). Hiatal hernia (OR, 5.9343; 95% CI, 2.6515 – 13.2816) and VAT (OR, 1.0074; 95% CI, 1.0001–1.0147) showed an independently significant association with the risk of BE, while the BMI and SAT showed no such association. In regard to the circulating factors secreted from adipose tissue, while the serum adiponectin level was significantly lower in the group with BE than in the group without BE (p = 0.0089), the serum leptin level tended to be higher in the group with BE than in that without BE, although the difference did not reach statistical significance (p = 0.5058) (Table 2). In other laboratory parameters, the only serum CHE level was significantly higher in the group with BE than in that without BE (p = 0.0225). In addition, the serum AST (p = 0.0789) and ALT (p = 0.0645) levels were higher in the group with BE than in that without BE, but the differences did not reach significant levels.

**Table 1:** Clinical characteristics of the patients enrolled in the present study.

Clinical characteristics	Number (%)
Total number of patients	163
	
Patient profiles	
Age: median; range (years)	60; 23–80
Sex: Female (%)	80 (49.1)
BMI: median; range	25.0; 19.2–43.6
	
Endoscopic results	
Hiatal hernia (%)	41 (25.1)
Erosive esophagitis (%)	
Total	34 (20.9)
Mild	29 (17.8)
Severe	5 (3.1)
Endoscopic BE (%)	
Total	69 (42.3)
SSBE	67 (41.1)
LSBE	2 (1.2)

BMI; body mass index, BE; Barrett's esophagus, SSBE; short-segment Barrett's esophagus, LSBE; long-segment Barrett's esophagus

**Table 2:** Clinical characteristics of the patients according to the presence or absence of endoscopic Barrett's esophagus.

	Endoscopic BE		
		
	Negative (n = 94)	Positive (n = 69)	P-value
Patients profiles			
Age			
median; range (years)	60; 23–80	60; 30–77	0.8825*
Sex			
Female (%)	48 (51.1)	32 (46.4)	0.5542**
BMI			
median; range	24.1; 19.2–39.7	25.8; 19.2–43.6	0.1354*
			
Endoscopic results			
Hiatal hernia (%)	11 (11.7)	30 (43.5)	<0.0001**
Erosive esophagitis (%)	13 (13.8)	21 (30.4)	0.0099**
			
Abdominal surface area			
VAT (cm^2^)	97.45 (19.9–514.2)	120.2 (27.6–398.8)	0.0089*
SAT (cm^2^)	158.15 (34.5–428.7)	180.4 (35.2–484.8)	0.3169*
			
Circulating factors secreted from adipocytes			
Leptin (ng/mL)	9.45 (2.5–27.6)	12.15 (2.7–26.4)	0.5058*
Adiponectin (μg/mL)	7.9 (3.2–21.4)	5.6 (3.2–12.5)	0.0089*
			
Other laboratory parameters			
AST (U/L)	24 (12–112)	27 (12–204)	0.0789*
ALT (U/L)	30 (4–198)	32 (11–258)	0.0645*
CHE (U/L)	341 (208–719)	372 (198–545)	0.0225*
HDL (mg/dL)	57 (23–106)	50 (28–93)	0.1158*
LDL (mg/dL)	125.5 (40–202)	125 (61–241)	0.7548*
TG (mg/dL)	120 (50–524)	145 (52–809)	0.1076*
HOMA-R	2.444 (0.578–26.929)	3.184 (1.102–13.743)	0.3538*

*Mann-Whitney *U *test, **Chi square test.

**Table 3:** Multiple logistic regression analysis to identify the clinical factors associated with a risk of endoscopic Barrett's esophagus.

Factor	Odds ratio	95% confidence interval	P-value
Age	0.9990	0.9681–1.0308	0.9489
Sex: Female	1.1361	0.5063–2.5490	0.7569
BMI	0.9880	0.8640–1.1298	0.8597
Hiatal Hernia	5.9343	2.6515–13.2816	<0.0001
VAT	1.0074	1.0001–1.0147	0.0472
SAT	0.9990	0.9932–1.0048	0.7377

The study population frequently had suffered from hypertension and taken Ca channel blockers, have the possibility of modification to LES pressure. But, no significant difference of prevalence of taking Ca channel blocker between BE group and non BE group (data not shown).

## Discussion

In the present study, 42.3% (SSBE, 41.1%; LSBE, 1.2%) of the study population was diagnosed as having BE based on the Prague C & M Criteria (Table 1). The findings are consistent with previous reports from Japan indicating that SSBE is more common, while LSBE is rarer as compared with the observations in the United States and Western Europe [29,30]. The frequency of BE might be affected by differences in its definition, in particular, by whether the presence of intestinal metaplasia is essential for making the diagnosis. The British Society of Gastroenterology guidelines propose that histological evidence of specialized intestinal metaplasia is not necessary for the diagnosis of BE, as its absence in one set of biopsies may be solely due to sampling error, and the tissue might still have an increased neoplastic potential as compared to that lined by squamous epithelium [31]. This is considered as a realistic recognition. In other western countries, the confirmation of the presence of intestinal metaplasia of the esophagus by biopsy is essential for making a diagnosis of BE [32], as it is considered as a risk factor for esophageal adenocarcinoma [33]. In this connection, in the present study, BE was diagnosed endoscopically based on the Prague C & M Criteria [28] without histological confirmation, and was therefore defined as endoscopic BE.

The results of univariate analysis in the present study conducted on Japanese patients with NAFLD showed that VAT was significantly associated with BE, whereas the association of the BMI with BE did not reach statistical significance (Table 2). Furthermore, the results of the multivariate analysis indicated that VAT was independently associated with the risk of BE, even after adjustment for the BMI and SAT (Table 3). El-Serag et al. reported, in a case-control study in which the majority of the participants were white subjects, that both BMI and VAT were significantly associated with an increased risk of BE, and that VAT remained independently associated with the risk of BE even after adjustment for BMI, while the significant association between BMI and the risk of BE was abolished after adjustment for VAT [15]. These findings suggest that the effect of obesity on the risk of BE is mainly mediated by abdominal obesity, especially the visceral fat area, rather than by simple obesity. The difference in the strength of association between BMI and the risk of BE between these two reports might be explained, at least in part, by ethnic differences in the obesity pattern, especially the pattern of visceral adipose deposition. Abdominal obesity can explain, in some part, the epidemiological features of BE and esophageal adenocarcinoma. For example, the body fat distribution tends towards visceral obesity than simple obesity in high-risk groups for BE, including Caucasians (as compared with Asian and African populations), and men (as compared with women) [34]. In addition, the increasing incidence of esophageal adenocarcinoma over the past two decades has been paralleled by an increase in the prevalence of obesity [35].

Several plausible mechanisms might explain the relation between abdominal obesity and the risk of BE and esophageal adenocarcinoma [36,37]. First, abdominal obesity can cause direct mechanical pressure on the stomach, increasing the intragastric pressure and leading to a higher frequency of relaxation of the lower esophageal sphincter and consequent reflux. Recently, although both BMI and WC have been reported to be associated with the intragastric pressure and the gastroesophageal pressure gradient, the WC has been reported to show a stronger association than the BMI [38]. Accordingly, abdominal obesity may be an important causative factor of GERD, as a major risk factor for BE. A second mechanism may be related to the metabolic activity of the adipose tissues, especially the visceral adipose tissue, which release adipokines, including IL-6 and TNF-α [21], that may play a role in the development of BE or the consequent carcinogenesis. Leptin is secreted predominantly by adipose tissues, and the serum levels of this adipokine increase in proportion to the body fat mass [22]. Leptin has been shown to stimulate cell proferation and inhibit apoptosis in Barrett's-derived EAC cells [23]. The characteristics of increased proliferation and reduced apoptosis, which are often noted in BE, are important for the progression to cancer, because they promote the accumulation and persistence of genetic abnormalities. Kendall BJ et al. reported a threefold increased risk of BE among men with serum leptin levels in the highest quartile, and that this increase in the risk persisted even after adjustment for the symptoms of GERD. In contrast, the risk of BE decreased with increasing serum leptin levels in women [24]. In the present study, the serum leptin levels tended to be higher in the group with BE than in that without BE, however, this relationship did not reach statistical significance (Table 2). The study population in this study might have been too small to allow examination of any gender difference in the association between the serum leptin and the risk of BE (data not shown). Adiponectin is a peptide secreted primarily from visceral adipocytes, and its serum levels are inversely associated with the degree of obesity. Adiponectin inhibits inflammation and promotes apoptosis, and deficiency of adiponectin has been implicated in a number of epithelial cancers [25]. Rubenstein JH et al. reported that deficiency of adiponectin was associated with the presence of BE, even after adjustment for the duration of GERD symptoms [26]. The present study showed a significant association between the serum adiponectin level and the risk of BE (Table 2), consistent with the results of the aforesaid study. Analysis of other laboratory parameters indicated that NAFLD tend to be more severe in the group with BE than in that without BE, the relationship did not reach the significant level.

The present study results suggest that visceral obesity may be involved in the risk of development of BE in Japanese patients with NAFLD. As visceral obesity is the core component of the metabolic syndrome, it may be considered that the metabolic syndrome is associated with an elevated risk of BE. Associations between BE and individual components of the metabolic syndrome other than obesity have not been clearly elucidated. Each component of metabolic syndrome may interact to increase the risk of BE; accordingly, a cluster of metabolic abnormalities that fulfill the diagnostic criteria of metabolic syndrome may increase the risk of BE to a greater degree than any individual metabolic abnormality. However, sufficient evidence has not been collected to prove this hypothesis, and further studies are needed to clarify these associations.

The present study rouse our interest in whether the same association between visceral obesity and the risk of BE may be drawn in patients with alcoholic liver disease (ALD). But, the exact confirmation of the association is thought to be difficult, because alcohol drinking habit is also significantly associated with BE.

Our study also had several limitations that need to be considered. First, only NAFLD cases were included in our study population. Whether our results would also be applicable to the general population remains to be determined. There is no doubt NAFLD patients tend to have more visceral obesity compared with the general population. Second, our study population was probably too small to allow examination of any gender differences in the associations of obesity parameters with BE. Because the prevalence of visceral obesity varies by gender [39], gender differences possibly exist in the associations between visceral obesity and circulating factors secreted from adipocytes and the risk of BE.

A major advantage of this study was the use of CT, which has a high degree of validity and reproducibility, to estimate the abdominal fat area. Abdominal fat may be subdivided into SAT and VAT. While CT allows direct assessment of these two fat compartments, anthropometric measurements (e.g., WC) do not [40]. The surface area of VAT measured in a single-cut CT scan taken at the level of the umbilicus has been shown to be a highly accurate and reproducible measure of the volume of VAT (correlation coefficient = 0.9) that may be calculated from 3-D image reconstruction of multiple CT or MRI cuts [41,42]. VAT has been extensively used as an indicator of the amount of abdominal fat, and has also been shown in case-control and cohort studies to be a strong predictor of the insulin resistance syndrome, diabetes, and coronary artery disease. To the best of our knowledge, no studies have been conducted until date to examine the effect of visceral obesity, as measured by calculation of the surface area of VAT, on the risk of development of BE in the Asian population.

## Conclusion

In summary, this study showed the existence of a significant association between visceral obesity, as measured on abdominal CT images, and the risk of BE in a Japanese population with NAFLD. This raises several questions regarding the pathogenesis of obesity-related GERD and its potential complications, including BE. Larger studies with prospective enrollment of patients are required for further examination of this issue.

## List of abbreviations

BMI: body mass index; WC: waist circumference; WHR: waist-to-hip ratio; VAT: visceral adipose tissue; SAT: subcutaneous adipose tissue; LA Classification: the Los Angeles Classification; BE: Barrett's esophagus; SSBE: short-segment Barrett's esophagus; LSBE: long-segment Barrett's esophagus; OR: odds ratio; CI: confidence interval.

## Competing interests

The authors declare that they have no competing interests.

## Authors' contributions

TA, TK, CT, AG, KK and MY analyzed the upper gastrointestinal endoscopies, collected the clinical data and wrote the manuscript, with contributions from MI. AN and MI was responsible for the design of the study and collected the clinical data.

HK, NK, SS, HI, KH and KY performed the statistical analyses. TA, MY, KF, HT, AY and MI analyzed the upper gastrointestinal endoscopies and participated in the design and coordination of the study. All the authors have read and approved the final manuscript.

## Pre-publication history

The pre-publication history for this paper can be accessed here:

http://www.biomedcentral.com/1471-230X/9/56/prepub
